# Development of a mobile phone camera-based transcutaneous bilirubinometer for low-resource settings

**DOI:** 10.1364/BOE.449625

**Published:** 2022-04-15

**Authors:** Brandon Harrison-Smith, Alexander P. Dumont, Mohammed Shahriar Arefin, Yu Sun, Nuradeen Lawal, Dorianna Dobson, Amy Nwaba, Sarah Grossarth, Abdulsalam Muhammed Paed, Zubaida L. Farouk, Jorn-Hendrik Weitkamp, Chetan A. Patil

**Affiliations:** 1 Department of Bioengineering, Temple University, 1801 N. Broad St., Philadelphia, PA 19121, USA; 2 Department of Pediatrics, Aminu Kano Teaching Hospital(AKTH), Kano 7002 31, Nigeria; 3 Department of Pediatrics, Vanderbilt University Medical Center(VUMC), Nashville, TN 37203, USA

## Abstract

Newborns in high-income countries are routinely screened for neonatal jaundice using transcutaneous bilirubinometery (TcB). In low-and middle-income countries, TcB is not widely used due to a lack of availability; however, mobile-phone approaches for TcB could help expand screening opportunities. We developed a mobile phone-based approach for TcB and validated the method with a 37 patient multi-ethnic pilot study. We include a custom-designed snap-on adapter that is used to create a spatially resolved diffuse reflectance detection configuration with the illumination provided by the mobile-phone LED flash. Monte-Carlo models of reflectance from neonatal skin were used to guide the design of an adapter for filtered red-green-blue (RGB) mobile-phone camera reflectance measurements. We extracted measures of reflectance from multiple optimized spatial-offset regions-of-interest (ROIs) and a linear model was developed and cross-validated. This resulted in a correlation between total serum bilirubin and mobile-phone TcB estimated bilirubin with a *R*^2^= 0.42 and Bland-Altman limits of agreement of +6.4 mg/dL to -7.0 mg/dL. These results indicate that a mobile phone with a modified adapter can be utilized to measure neonatal bilirubin values, thus creating a novel tool for neonatal jaundice screening in low-resource settings.

## 1. Introduction

Neonatal jaundice (NNJ) is a common condition clinically associated with some degree of yellowing of the skin and eyes that typically self-resolves in the first week after birth [1]. NNJ is characterized by an elevation of serum bilirubin levels driven by underdeveloped bilirubin metabolism. However, prolonged and extreme hyperbilirubinemia (EHB) is problematic due to bilirubin’s neurotoxicity and can ultimately lead to kernicterus, severe neurosensory deficit and even death [2].

EHB-related neonatal mortality in low- and middle-income countries (LMIC) is reported to be approximately 638 infants per 10,000 births, compared to 3.7 infants per 10,000 births in high-income countries [3]. In LMIC, poor EHB-related outcomes arise from a multitude of factors, including increased underlying prevalence of genetic conditions such as glucose-6-phosphate dehydrogenase (G6PD) deficiency and hemolytic diseases, increased prevalence of risk factors such as sepsis and low birth weight, high-rates of non-institutional delivery resulting in delayed care, as well as inconsistent availability of laboratory based diagnostic testing and phototherapy treatment units [4–7]. While the clinical reference for diagnosis is gold-standard blood testing of total serum bilirubin (TSB), inconsistent availability of facilities and supplies remain a problem due to high rates of home births in LMIC. Therefore, simple visual examination of skin blanching is used for newborn jaundice screening, despite limited precision and inferior ability to predict neurological injury from EHB in comparison to TSB [8–9]. Low-cost point-of-care blood testing solutions can potentially reduce barriers to quantitative assessment of bilirubin that exist in LMIC [10–13]; however, a non-invasive approach could be a desirable alternative for early screening.

Transcutaneous Bilirubinometers (TcB) are a widely used optical reflectance technique that performs screening measurements near bilirubin’s absorption maxima at 460 nm along with at least one additional reference measurement above 500 nm to normalize for optical variations in tissue including perfusion, pigmentation and scattering [14], while also detecting two distinct spatially offset detection channels with different depth-dependent biases [15]. Over the last 20 years, TcB has become an important element in a systematic approach for universal screening newborn’s risk for EHB in high-income countries [16–18]. Infants identified as at-risk subsequent diagnosis can be confirmed via TsB, appropriate treatments are administered in a timely fashion, and instances of chronic bilirubin encephalopathy (CBE) and EHB-related deaths are extremely rare in high income countries such as the United States [3,19]. Expanded adoption of TcB in LMICs has been proposed but the cost of currently available TcB devices remains an obstacle. As a result, development of low-cost and mobile phone-based approaches to estimate serum bilirubin levels have been proposed [20–22]. In particular, a mobile phone-based platform offers a strong potential for integration with communications networks for coordination of care, healthcare data management, and real-time patient monitoring [23–25]. In this manuscript, we report the development of a mobile phone-based TcB device. The approach mimics clinical TcB by directly measuring a spatially-offset diffuse reflectance in direct contact with the infant’s skin. Monte-Carlo models of reflectance from neonatal skin were used to guide the design of an adapter for filtered Red-Green-Blue (RGB) mobile phone camera reflectance measurements. A pilot study was performed across two sites in the United States and Nigeria in order to include infants with a wide range of pigmentation; and a generalized linear model was developed using multiple spatial offset regions-of-interest (ROIs) across the RGB reflectance channels. Results indicate that this approach holds promise for mobile phone-based approaches for estimating bilirubin in neonates. While there are multiple mobile phone based TcB devices, no devices to our knowledge have been shown to utilize multi-band filters in conjunction with a smart ROI search system and include data from African neonates.

## 2. Materials and methods

### 2.1 Monte-Carlo modeling of a smartphone based TcB device

The principle of the mobile phone TcB device reported here is inspired directly from clinical TcB. Monte-Carlo (MC) modeling of photon migration in tissue has been a well-suited approach for theoretical investigation of diffuse reflectance in TcB, and has provided valuable insights into the influence of pigment, scattering, illumination/collection geometry and light source selection [26,27].

**Fig. 1.**
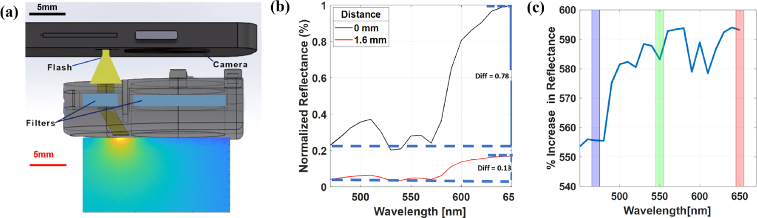
(a) 3D physical model of the mobile phone TcB design which depicts illumination through the device. An MC photon fluence map depicts modeled light distribution in neonatal skin. (b-c) Monte Carlo simulation results (b) Normalized reflectance for two configurations (c) Percent increase of reflectance from 0mm to 1.6mm configurations in the red, green and blue filtered channels.

The overall design concept was to create an adaptor that could be placed over the phone’s LED light source and camera module. Monte Carlo simulations were used to guide illumination/collection geometry design and optimize the collection of a wide range of reflectance intensities observed when capturing spatially resolved images at multiple spatial reflectance offsets seen across the 400-700 nm spectral range. Our preliminary data suggested a large mismatch in reflectance across the blue and red channels leading to an insufficient blue channel reflectance signal at appropriate spatial offsets [28]. We created MC models for diffuse reflectance of neonatal skin with illumination/collection parameters that matched the proposed geometry of the mobile phone camera adaptor, and then used the simulation outputs to determine the relative benefit that a reduction in source-detector separation would have on improving disparities expected in red/blue channel reflectance. A physical design of the prototype smartphone adaptor, along with a representative MC simulation of photon flux in the skin, is shown in Figure 1(a).

Monte-Carlo modeling was performed using the Monte Carlo eXtreme (MCX) platform [29,30]. A generalized tissue optical model was configured to simulate neonatal skin, with parameters related to skin thickness, scattering, and chromophore concentration and extinction informed from prior work [27,31]. Melanosome fractional volume was set at 10% to determine model performance in darkly pigmented skin, where overall signal intensities are lower [27,28]. The optical illumination and detection configuration in MCX was directly informed by both the physical and optical specifications of the LG Nexus 5 Android smartphone and the proposed adaptor, including LED-to-camera offset edge-to-edge offset, illumination spot size, detector size and imaging optics. Two optical configurations were simulated; (1) the offset between the proximal edge of the illumination beam and the proximal edge of the camera field-of-view was set to 1.6 mm, and (2) the offset was reduced to 0 mm. In both configurations, the adaptor thickness was set to 10 mm, and modeled spectral diffuse reflectance from 400-700 nm was determined based on inclusion of all diffusely reflected simulated photons within the acceptance angle of the imaging optics arising from within the 9mmx12mm field of view (FOV). FOV was verified through empirical measurements with a microscopy calibration grid. Thus, the source-detector offset from the center of the source to the center of the image FOV for the two configurations is 7.5mm and 9.1mm. After running the MC simulations, modeled spectral normalized diffuse reflectance is generated for the two configurations (Figure 1(b)), and the % reflectance Increase differences in spectral intensities were compared across each RGB color channel (Figure 1(c)). Results of these simulations indicate differences in the relative magnitude of color-channel dependent changes in detected reflectance for the two flash-detector offsets, and informs any potential reduction in the range of reflectance values measured across channels.

### 2.2 Mobile phone TcB optical adapter

**Fig. 2.**
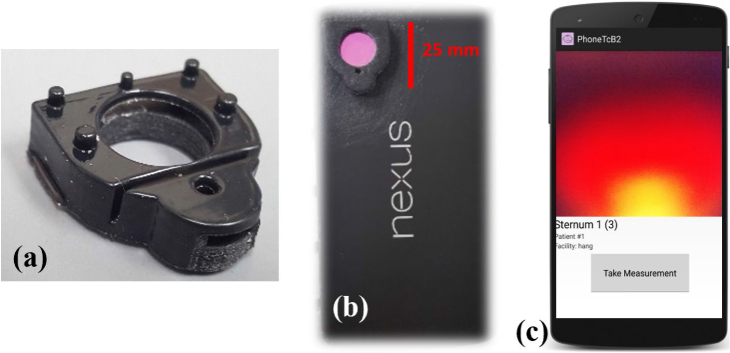
Implemented Smartphone-Based TcB Device: (a) 3D printed adapter which guides light towards skin and house the band-pass filters (b) Device attached to the phone casing with filters (c) Smartphone application interface while taking a measurement from skin.

Based on the results of the MC models, an adaptor (shown in Figure 2(a)) was developed to be placed between the mobile phone (LG Nexus 5) camera/LED and the infant’s skin, with the purpose of redirecting LED illumination to a confined spot with minimal spatial offset from the camera’s field of view and housing triple-band pass optical filters for improved spectral isolation of RGB measurements (Figure 1(a)).

A 10 mm thick prototype adapter was 3D printed (Connex 3 Object 500 3D printer, Stratsys Ltd.) using black material (Vero Black, Stratsys Ltd.) in order to reduce the intensity of light that may transmit through the plastic. The adapter (Figure 2(a)) was fixed to a generic snap-on protective case and aligned over the camera and flash. Although the 10 mm thickness of the adapter is shorter than the minimum working distance, a measurement of the defocused edge response resulted in a 90% to 10% width corresponding to approximately 1.4 mm over the 12 mm image width. A 2mm pinhole on the phone-facing side of the adapter served to reduces the half angle of the LED from approximately 41 degrees to 21 degrees to preserve the spectral performance of a triple-band pass filter (Idex Semrock FF01. D=5mm) with passbands at 474 +/- 10nm (blue), 554 +/- 10nm (green), and 635 +/- 10nm (red). After the filter, an angled 2mm channel directs the light towards the camera field of view, with a minimum offset that is limited by performance of the 3D printer. The spatially offset diffuse reflectance is then collected via a separate optical path that includes a notched space for a second, larger triple-band pass filter (D=10mm) with identical spectral profile. The adapter containing band-pass filters attached with the smartphone is shown in Figure 2(b). All components, including the 3D print material and filters were selected based on their resistance to degradation from simple disinfectant with 70% ethanol protocols [32,33].

### 2.3 Diffuse reflectance measurements and calibration

A custom mobile application was developed in order to configure mobile phone image acquisition, as well as guide users through calibration and clinical measurements. The basic interface of the smartphone application during image capturing is presented in Figure 2(c). Image data is configured to be saved without pre-processing or compression in raw 16-bit format in order to ensure the data could be used for quantitative measurement of reflectance. Camera acquisition parameters were fixed, including focus, exposure time and ISO/gain. Each measurement consisted of acquisition of 3 successive tissue reflectance images with exposure time of 400ms and ISO of 4500, along with a paired set of 3 dark background images collected with the LED off. In this study, 3 replicate sets of measurements were collected from each infant to ensure unexpected clinical scenarios, such as sudden movements, did not reduce enrollment yield.

Calibration was performed by measuring a custom calibration standard consisting of a 25x25x5(thick) mm High-Density Polyethylene (HDPE) white plastic block housed in a Vero Black casing using an identical sequence of reflectance (LED on) and background (LED off) image acquisitions. Paired calibration measurements were collected for each patient enrolled.

### 2.4 Human subjects

**Fig. 3.**
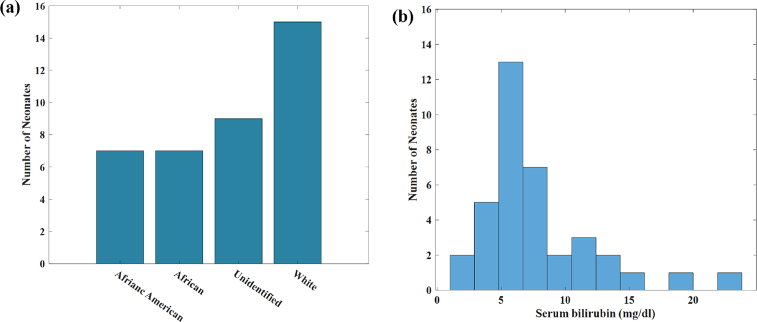
Human Subject Study: (a) Number of neonates used in the study (b) Serum bilirubin distributions of neonates where majority of the TSB values are clustered around 5-10 mg/dL.

Measurements were collected utilizing the mobile phone TcB device from 37 neonates (30 from Vanderbilt University Medical Center (VUMC), Nashville, TN, USA, and 7 from Aminu Kano Teaching Hospital (AKTH), Kano, Nigeria). Healthy infants between 24-72 hours postnatal age were considered eligible for this study. Exclusion criteria included infants born with known or estimated gestational age < 32 weeks, weighed under 1500 grams, or prior phototherapy. Each mobile phone measurement was obtained within 30 minutes of a blood sample used to obtain TSB. Written consent was obtained from all parents, and all studies were performed in accordance with the institution’s human subjects research approvals (VUMC IRB130471 and AKTH EC1390). All measurements were collected using the smartphone in direct contact with the sternum of the infant while minimizing in rooms where bright ambient lighting was minimized. Our device requires that all measurements be made with the device placed flat and flush against the skin. The forehead has varying curvatures between neonates, while the sternum location has less variability in curvature. This allows for more uniform and consistent measurements taken with our device. The demographic distributions of the neonates are provided in Figure 3(a). The distribution of TSB values obtained for all the neonates are shown in Figure 3(b), indicating a right-skewed, non-normal distribution characteristic of newborn TSB, with a majority of values between 5-10 mg/dL, and some infants with elevated values as high as 23.7 mg/dL.

### 2.5 Image pre-processing

**Fig. 4.**
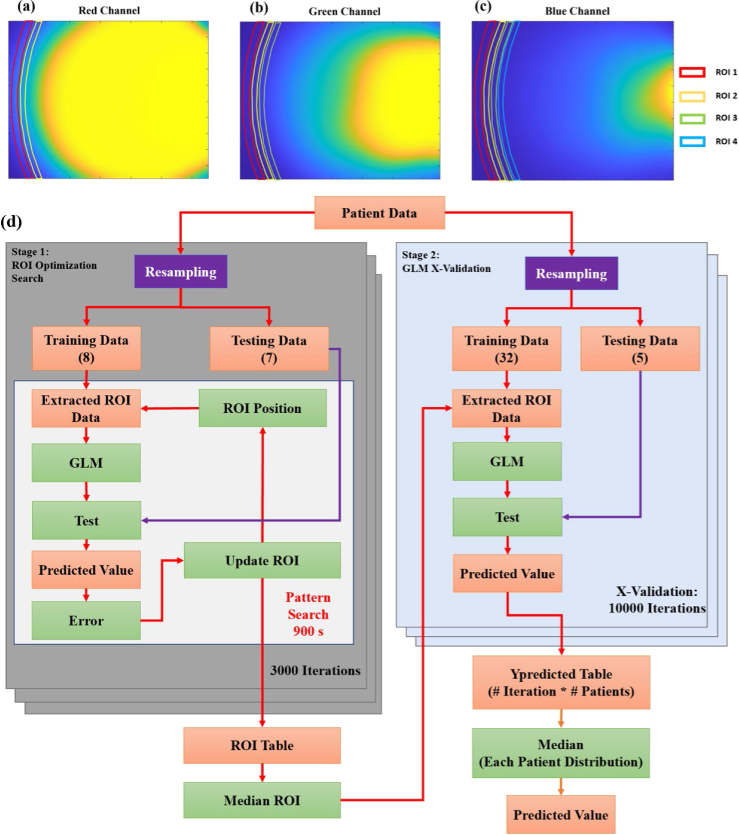
Two-Stage ROI-based GLM. Images and ROI initial positions for (a) Red (2 ROIs),(b) Green (3 ROIs), and (c) Blue (4 ROI’s) channels. (d) The overview of the two-stage ROI optimization and model cross-validation algorithm. Input, intermediate, and output data depicted as orange boxes; primary operations as purple boxes, and secondary operations as green boxes.

Raw images were extracted from the mobile device and converted to Digital Negative (.dng) format with an Adobe DNG Converter prior to import in MATLAB (Mathworks) for processing. DNG images were demosaiced, resulting in Red, Green and Blue (RGB) and resized to a quarter of the original image to reduce the data size for analysis. All images were processed through a multivariate k-nearest-means outlier detection algorithm [34], which was used to identify sets of images that contained poorly performed measurements (i.e. infant motion, device non-contact). Once the outliers were removed, each set of images (sternum and calibration images) was averaged, resulting in one final image each for sternum reflectance, sternum background, calibration reflectance and calibration background was associated with each patient. Reflectance images were background subtracted and normalized by calibration images in order to produce a single RGB-calibrated reflectance image for each patient. Representative patient RGB image data used for subsequent analysis is shown in Figure 4(a-c).

### 2.6 Development of region of interest (ROI) based generalized linear model

Optical estimation of bilirubin levels is classically informed through creation of linear regression models of the reflectance from unique spectral and spatial channels against matched TSB values [14]. In our mobile phone TcB device, the pre-processed images represent a spatially resolved map of tissue diffuse reflectance, and thus a linear model can be created similar to classical TcB using multiple unique spatial offset regions-of-interest (ROI) within each RGB spectral image channel. Unlike conventional TcB, where physical detectors placed at unique offsets define independent spatial channels, collection of images of diffuse reflectance produces a spatially resolved reflectance map and offers the opportunity to define multiple spatial offset channels uniquely in each RGB spectral channel. In order to determine appropriate placement of the spatial offset channels and to develop the corresponding linear model, a two-stage approach was used. In the first stage, a bounded pattern search optimization algorithm was used to identify the placement of specific spatial offset channel’s regions-of-interest (ROI). In the second stage, the reflectance within the ROI’s was used to develop and cross-validate a generalized linear model (GLM) to estimate TSB. A block diagram depicting two-stage model development and cross-validation is shown in Fig. 4(d).

Based on the right-skewed non-negative distribution of the TSB values (Figure 3(b)) along with the resulting limited number of infants enrolled with TSB values > 15 mg/dL, fully randomized selection of patients to include in training subsets would result in high likelihood models constructed in individual cross-validation iterations were informed with data from infants with TSB tightly clustered between 5-7 mg/dL, and oftentimes no subjects > 12 mg/dL, while the clinically relevant range of newborn bilirubin levels from 0-20 mg/dL.

In order to account for this enrollment-dependent limitation in the ROI optimization stage, the full data set was first sorted according to TSB values, and then divided into 4 different ranges (0-5mg/dl, 5.01-10 mg/dl, 10.01-15mg/dl and 15.01-25 mg/dl). Training data sets consisted of randomized resampling of 2 patients each across the 4 TSB bins to total 8 uniformly sampled patients across the full TSB range. The ROI test sets were then selected from the remaining pool of patients with 2 patients from the first 3 ranges and the 1 remaining patient left in the highest TSB range (only 3 enrollees had TSB levels > 15 mg/dL) to total 7 patients. This resampling approach ensured uniform distributions to guide ROI optimization despite limitations in the sample sets distribution. A bounded pattern search algorithm implemented in the MATLAB optimization toolbox (Mathworks) was used to optimize ROI placement [35]. Here, the spatial offset areas are arc-shaped regions-of-interest (ROI) in each spectral (RGB) image frame defined based on their position, radius and width. Initial conditions for 4 ROIs in the blue channel, 3 ROIs in the green, and 2 ROIs in the red (Figure 4(a-c)).The number of ROI’s per channel was selected based on the relative amount of unsaturated area in each image channel’s FOV, where the blue channel had the most, and the red channel had the least. The pattern search algorithm uses the mean values within the ROIs as the observed variables for a gamma distribution (non-negative constraint) based GLM [36] to predict TSB. The pattern search algorithm aims to iteratively adjust the positions of the ROIs in order to optimize the sum square error of the GLM over a duration of 900s. After completion of the pattern search, the ROIs are stored in a table, and the optimization was repeated over 3,000 different resampled iterations, resulting in 3000 different estimates of ROI positions. The median of each ROI position was calculated to determine the 9 different ROIs which were served as the output of stage 1, and were used for further analysis. After the final ROI positions were determined, the second stage of the routine performed cross-validation of the GLM’s ability to predict the bilirubin values from patient images. Here, the patient images were sorted by bilirubin values and divided into 5 different ranges (0-3.5mg/dl, 3.51-6.5 mg/dl, 6.51-10 mg/dl, 10.01-15mg/dl and 15.01-25 mg/dl). In each cross-validation, 1 patient was selected randomly from each of the ranges to create a 5 patient test set. The remaining 32 patients were considered for the training set. Using the median ROI positions measured from the initial pattern search-based algorithm, ROI data were extracted from the training data to create a GLM. The GLM’s performance was evaluated on the testing dataset. This process was carried out for 10000 cross-validations. After running 10000 cross-validation, a table of predicted values was generated containing a distribution of predicted values for each patient. Then, the median of each patient’s predicted values was taken. Cross-validation using this approach for resampling training and testing data over many iterations [37,38] can then be used to produce a robust composite estimates of the model’s performance.

**Fig. 5.**
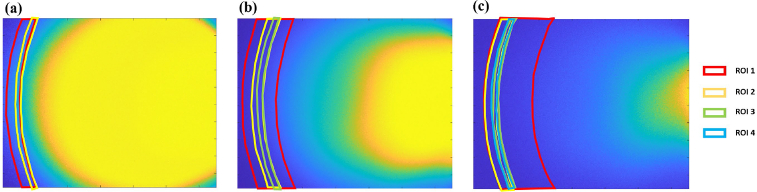
Images with final ROI positions for (a) Red (2 ROIs),(b) Green (3 ROIs), and (c) Blue (4 ROI’s) channels generated after stage 1 ROI optimization.

A correlation plot for predicted bilirubin values vs. TSB and a Bland-Altman plot were generated to report model performance, bias and limits of agreement. In addition, the final ROI parameters were calculated as the mean positions across all cross-validations. The final median ROIs calculated across all cross-validations are presented in Figure 5(a-c).

## 3. Results

### 3.1 Monte Carlo model

A MCX model was used to compare the effect of reducing the illumination-detector offset on the collected diffuse reflectance signal. Figure 1(b-c) represents the impact of source detector offset on diffuse reflectance intensities across the spectral range. In Fig. 1(b) there is a clear increase in measured reflectance when the source to detector separation is decreased from 1.6mm to 0mm. In addition, the modeled spectra indicate blue channel reflectance to be the lowest, while red channel reflectance to be the highest. The difference between reflectance intensities from blue to red is calculated as 0.13 for the 1.6 mm configuration vs. 0.78 for 0 mm configuration. Figure 1(c) also shows percent increase in the reflectance for the 0 mm vs. the 1.6 mm configuration. Note that while reducing the offset increases the weak blue channel reflectance, it results in a slightly greater increase in the red channel.

### 3.2 Mobile phone TcB design and characterization

A prototype and implemented mobile phone TcB device is illustrated in Fig. 1(a) and Fig. 2(b). The Mobile Phone TcB device is directly mounted over the flash and camera of the LG Nexus 5 mobile phone. Along with the adapter, the mobile phone TcB app is used to help users capture images of the neonate’s skin. The light from the flash is then filtered and enters the skin at a 30 degree angle with a 0 mm source to detector separation.

### 3.3 Pilot study results

**Fig. 6.**
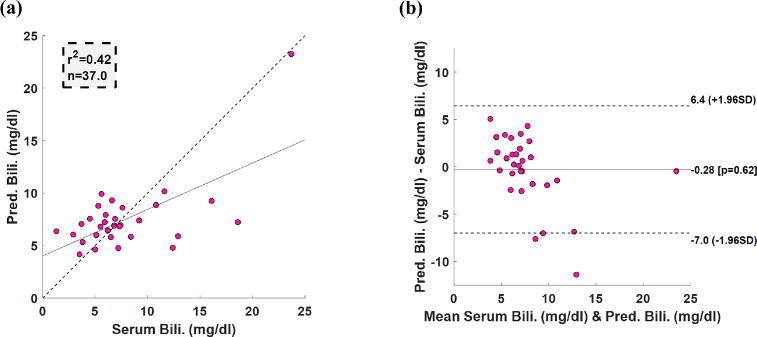
Linear regression statistics (a) Correlation(b) Bland-Altman of the mobile phones predicted bilirubin values vs. TSB values (n=37 patients).

The ability to measure bilirubin levels was evaluated in a pilot study conducted at Vanderbilt University Medical Hospital (VUMC) and Aminu Kano Teaching Hospital (AKTH). Figure 3(a) represents a demographic breakdown of the 37 neonates that participated in this study. There are 14 African or African American neonates , 15 Caucasian neonates and 8 race unclassified neonates. TSB and mobile phone TcB measurements were collected from each patient. The measured bilirubin TSB levels complemented the clinical bilirubin range from 0-25mg/dL, with the majority ranging between 5 and 10 mg/dL. Figure 6(a) shows a correlation for the predicted TSB levels from the mobile phone TcB device with an $r^{2}$ value of 0.42 ( $p=1.41*10^{-5}$ ). The Bland-Altman plot in Fig. 6(b) shows a mean difference of -0.28 mg/dL ( p=0.62 ) between the mobile TcB device and TSB measurements, and 95% Limits of Agreement (LOA) are 6.4 mg/dL to -7.0 mg/dL (range between the upper and lower limits of LOA equal to 13.4 mg/dL). The root-mean-square error (RMSE) for the range between 0-10 mg/dl is around 2.13 mg/dl, whereas the RMSE in the range between 10.01-25 mg/dl is 6.05 mg/dl. This could be since the dataset had fewer neonates over 10 mg/dl to create the linear model and test it. These results indicate the ability of the smartphone-based TcB device to estimate TSB values in a racially diverse group of neonates.

The root-mean-square errors (RMSE) for 3 different skin groups (light or dark skin) were calculated from the resulted predicted values. In light skinned infants(n=15) the RMSE was 4.77 mg/dL , whereas, in case of dark skinned neonate’s (n=14) the RMSE was 2.05 mg/dL . However, a two sided t-test showed that error in dark skinned infants is not significantly different than white infants.

## 4. Discussion

Extreme hyperbilirubinemia (EHB) contributes up to 14% of neonatal deaths and causes significant amount of neurological sequealae in low-resource settings [8]. Reducing the global burden of EHB in LMIC is a complex problem, including challenges in the lack of genetic screening, maternal education, adoption of universal practices for newborn screening and expansion of availability of phototherapy [39]. A low-cost mobile phone-based approach to TSB estimation can take advantage of the expanding access to smartphones across low-resource and rural areas in LMIC [40] and has substantial potential to improve screening practices if effective. Moreover, the connection of data and information services through smartphones offers the potential for coordination of treatment services for jaundiced infants requiring care in remote areas. Here we describe the development of a mobile phone-based approach for TcB measurement that uses an optical adapter fitting commercially available protective mobile phone cases. The adaptor design enables collection of spatially resolved images of diffuse reflectance with an offset illumination spot through a set of optical filters. The total cost of the adaptor, not including the mobile phone, is approximately less than $ 1,000 US dollars (Filters $ 400 US dollars each and 3D printed material: $ 20 US dollars). The cost of the commonly used Drager Jm-105 Jaundice meter can be priced at $ 1,100 US dollars. Although the filters used in this adaptor are scientific grade coated glass substrate, low-cost, durable, coated plastic optical filters allow production of similar adaptors for less than $ 50 US dollars [41]. Alternatively, spectral filters with reduced transmission in the red channel could be used to reduce the difference between reflectance from blue and red spectral channels to expand the spatial range of usable data by exposure balancing. The use of a passive adaptor, in combination with the novel approach of extracting multiple spatial and spectral ROI reflectance channels within the RGB image data, offers a promising and unique approach for TSB estimation in low-resource settings.

The mobile phone-based approach reported here produced an estimation of TSB within 6.4 to -7.0 mg/dL, with a bias of -0.28 mg/dL (Figure 6(b)). These results are comparable to, but not as positive as, previously published results of TcB-TSB agreement in large, multi-ethnic study populations, which have a maximum span of the reported LOAs falling between approximately 4 and -6.0 mg/dL and biases between 1.6 and -0.8 mg/dL [42–45]. This suggests promise that with further development, mobile-phone based approaches could evolve as a TcB surrogate.

An important issue reported in clinical TcB devices is both increased error and a systematic overestimation bias in black African neonates (LOA of 6.7 to -0.65 mg/dL with a bias of 3.04 mg/dL) [46]. TcB nomograms constructed using only black African neonates [47] have been shown to address these issues, indicating these problems may arise from skewed racial composition in enrollment of subjects during construction of the TcB regression models. Enrollment in this pilot study was nearly even between those of African-origin and White infants in an attempt to ensure enrollment biases minimally contribute to model construction. While the overall bias of this study is slightly negative, these pilot results require more robust statistical assessment of error and bias would require a larger scale study. It is worthwhile noting the results from this pilot study do not indicate either increased error of the over-estimation bias previously seen using TcB for black and African infants.

The fundamentally unique approach used in this study lies in the extraction of multiple individual spectral and spatial offset ROIs from filtered RGB images of spatially-offset diffuse reflectance. While clinical approaches to TcB measure diffuse reflectance in up to two different fixed spatial offset channels using between 2 and 5 wavelength channels to create a model for bilirubin estimation [15], the approach described here utilizes 9 uniquely optimized spatial ROIs across 3 different wavelength channels to inform a GLM [31]. Alternatively, the collection of multiple color-calibrated photographs of neonatal skin [22] have also been reported for accurate estimation of bilirubin levels (LOA of 3.6 to -3.6 mg/dL with 0.0 mg/dL bias) using algorithms not directly inspired by clinical TcB. In addition, measurement of neontal skin using a mobile-phone camera dermatoscope has also reported the ability to correlate with TSB in Caucasian infants (r^2^=0.81) [20]. It is possible that the differences in measurement techniques may produce different TSB estimation characteristics; although, further studies would be necessary. An important limitation of this pilot study is the relatively low number of enrolled infants with respect to the naturally skewed, non-normal, nature of newborn TSB distributions. A larger scale study with increased enrollment of infants with elevated TSB levels would be beneficial and reduce the need for resampling and large scale cross-validation to produce a generalized model.

Selection of an appropriate acquisition time that would allow proper exposure of the spatial decay of spatially offset diffuse reflectance across the RGB color channels represented a challenge for measurement configuration. The approach reported here requires configuring the camera hardware to collect the raw, uncompressed, 16-bit output of the detector array, as opposed to a JPEG compression, in order to retain the ability to perform absolute quantification of reflectance and preserve the available dynamic range of the detector. The more than 2-fold difference in the red channel to blue channel reflectance coupled with the requirement for large, uncompressed raw image data motivated the selection of a single exposure time which served as a compromise. Thus, this allowed the collection of maximal signals in the blue channel while still retaining a limited range of un-saturated reflectance in the red channel. Monte-Carlo models of reflectance in neonatal skin were used to investigate the benefit of altering source-detector offset in order to increase blue channel reflectance. Alternative strategies such as the collection of multiple raw images at different exposure times for high-dynamic range compression represents a promising future strategy which may improve the spatial range of unsaturated data available to place ROIs. While this may improve the TSB prediction model, collection of such large file size datasets was not feasible in this study.

The wide variety of mobile device models presents a challenge for many techniques developed atop mobile phone camera platforms. The technique we described would require the re-design of the adapter module that prioritized a similar field-of-view and illumination spatial-offset. Additional calibration of spectral throughput would also likely be necessary and could be performed using using the same calibration measurements performed in this study.

## 5. Conclusion

The availability, accuracy, and cost of current TcB devices remains an obstacle for their adoption within low-resource settings and LMICs as part of standard practice. The results presented here show that a mobile phone camera based TcB device using a simple, passive, adaptor and unique algorithm for extraction of multiple spatial and spectral ROIs from images of spatially-offset reflectance holds potential for estimating TSB, however additional developments will be necessary before the technique may prove valuable in LMIC settings.

## Data Availability

Data underlying the results presented in this paper are not publicly available at this time but may be obtained from the authors upon reasonable request.
